# Infosomes as Inflammatory Mediators: Proteomic Profiling of Proteins Enriched in Inflammatory Extracellular Vesicles

**DOI:** 10.1016/j.mcpro.2026.101511

**Published:** 2026-01-19

**Authors:** Semin Lee, Minjun Kim, Seungmin Lee, Hyo-Jin Kim, Ki-Jun Ryu, Sang-Hun Kim, Hong-Yeoul Ryu, Kyunghee Lee, Kwang Dong Kim, Jiyun Yoo, Cheol Hwangbo, Yong-ho Choe, Seongchan Kim, Seung Pil Yun, Hyuk-Kwon Kwon

**Affiliations:** 1 Division of Applied Life Science, Gyeongsang National University, Jinju, Republic of Korea; 2 Division of Bio & Medical Bigdata Department (BK4 Program), Gyeongsang National University, Jinju, Republic of Korea; 3 Research Institute of Life Sciences, Gyeongsang National University, Jinju, Republic of Korea; 4 Division of Life Science, Gyeongsang National University, Jinju, Republic of Korea; 5 Plant Molecular Biology and Biotechnology Research Center (PMBBRC) and Research Institute of Life Sciences, Geongsang National University, Jinju, Republic of Korea; 6 Department of Biochemistry, College of Medicine, Institute of Medical Science, Gyeongsang National University, Jinju, Republic of Korea; 7 Section of Pulmonary, Critical Care and Sleep Medicine, Department of Internal Medicine Yale School of Medicine, New Haven, Connecticut, USA; 8 BK21 FOUR KNU Creative BioResearch Group, School of Life Sciences, College of Natural Sciences, Kyungpook National University, Daegu, Republic of Korea; 9 High-Tech Materials Core Facility, Gyeongsang National University, Jinju, Republic of Korea; 10 Division of Applied Life Science (Brain Korea 21 Four), Research Institute of Life Sciences, Gyeongsang National University, Jinju, Republic of Korea; 11 Department of Veterinary Obstetrics, College of Veterinary Medicine, Gyeongsang National University, Jinju, Republic of Korea; 12 College of Pharmacy and Research Institute of Pharmaceutical Sciences, Gyeongsang National University, Jinju, Republic of Korea; 13 Department of Pharmacology, Institute of Medical Sciences, College of Medicine, Gyeongsang National University, Jinju, Republic of Korea; 14 Department of Convergence Medical Science, College of Medicine, Gyeongsang National University, Jinju, Republic of Korea

**Keywords:** extracellular vesicles, inflammation, infosomes, NLRP3 inflammasome, proteomics

## Abstract

Extracellular vesicles (EVs), including exosomes and microvesicles, act as transmitters of various biological signals through cell-cell communication. Although EVs derived from immune response cells have been partially studied, the characteristics of EVs mediated by NLR family pyrin domain-containing 3 (NLRP3) inflammasome activation remain unclear. Here, we characterize inflammatory EVs, termed infosomes, derived from NLRP3 inflammasome-activated macrophages, which play a role in inducing inflammation. Proteomic analysis revealed that EV production was increased in macrophages with activated NLRP3 inflammasomes and that these EVs were enriched with marker proteins involved in metabolism, membrane structure, and cytoskeletal organization. Furthermore, significantly increased proteins were associated with signaling pathways and biological processes related to immune response, phagocytosis, endocytosis, and neurodegenerative diseases. Crucially, these alterations in EV secretion and molecular composition were dependent on NLRP3 and its subsequent inflammasome activity. Functionally, these infosomes were shown to amplify the expression of inflammatory factors in both macrophages and endothelial cells. These findings provide insights into the biological roles of infosomes, suggesting that EVs generated and loaded by NLRP3 inflammasome activation act as key biological mediators that disseminate and amplify inflammatory responses through cell-cell communication. This highlights their potential as novel biomarkers and therapeutic targets for inflammatory diseases.

## Highlights

• Activation of the NLRP3 inflammasome enhances infosome secretion.
• Infosomes are enriched in proteins for immunity, neurodegeneration and EV transport.
• Infosomes are generated in an NLRP3-dependent manner.
• Infosomes induce inflammation in macrophages and endothelial cells.

## Graphical Abstract

**Figure:**
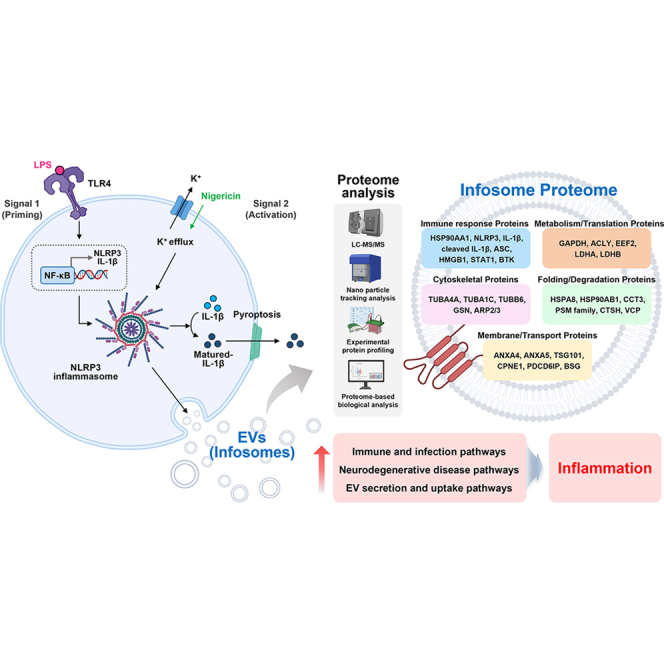

## In Brief

This study reveals that activation of the NLRP3 inflammasome triggers the release of inflammatory extracellular vesicles referred to as “infosomes”, which are characterized by a distinct protein profile and are capable of inducing inflammatory responses in recipient cells.

Extracellular communication is fundamental to immune regulation, enabling cells to coordinate responses to infection, metabolic changes, cellular senescence, and inflammation (1, 2, 3). A key mediator of this process is the Extracellular vesicles (EVs), a lipid bilayer-enclosed structure that facilitates the transfer of proteins, lipids, and nucleic acids, including RNA and DNA, between cells. The composition and function of EVs are largely determined by the physiological state of the donor cells, allowing these vesicles to participate in diverse biological processes (4). Increasing evidence suggests that EVs play a crucial role in immune modulation, particularly in inflammatory diseases, where they contribute to immune cell activation, tissue damage, and disease progression (5). However, the molecular characteristics and functional implications of EVs released upon innate immune activation remain poorly understood.

One of the key regulators of inflammation is the NLR family pyrin domain-containing 3 (NLRP3) inflammasome, a cytosolic multiprotein complex that detects pathogen-associated molecular patterns, damage-associated molecular patterns (DAMPs), and cellular stress (6, 7). Upon activation, NLRP3 recruits apoptosis-associated speck-like protein containing a CARD (ASC) and caspase-1, leading to the proteolytic cleavage and secretion of interleukin-1β (IL-1β) and interleukin-18 (IL-18) (8, 9, 10, 11). This cascade results in the amplification of inflammatory responses and can induce pyroptotic cell death. Excessive or unresolved inflammatory responses driven by innate immune activation have been implicated in the pathogenesis of various inflammatory diseases, including infection-associated conditions such as septic arthritis (12, 13, 14, 15, 16), as well as neurodegenerative disorders (17). While the role of NLRP3 in inflammatory disease progression has been well established, its influence on extracellular communication through EVs remains largely unexplored.

Recent studies suggest that EVs function as mediators of inflammation, facilitating the intercellular transfer of bioactive molecules that propagate immune responses beyond the initially activated cells (18, 19). EVs derived from activated macrophages have been shown to induce pro-inflammatory signaling in recipient immune and endothelial cells, contributing to chronic inflammation (20). Additionally, EVs transport pathogen-associated molecular patterns, DAMPs, cytokines, and miRNAs, which enhance inflammatory signaling pathways and promote tissue destruction in autoimmune diseases such as juvenile idiopathic arthritis, rheumatoid arthritis, and systemic lupus erythematosus (21). Despite these findings, it remains unclear whether activation of the NLRP3 inflammasome leads to the production of distinct EV subpopulations with specific molecular and functional properties and, if so, how these inflammasome-associated EVs contribute to disease pathogenesis.

To address this gap, we identify and characterize a novel subset of inflammatory EVs, termed infosomes that are secreted by macrophages upon NLRP3 inflammasome activation. Using quantitative proteomic analysis, we demonstrate that infosomes exhibit a distinct molecular composition in comparison to conventional EVs, being enriched in immune signaling proteins, cytoskeletal components, and regulators of the endocytic pathway. Notably, infosomes incorporate key immune response proteins, including the NLRP3 inflammasome, and their uptake by recipient cells induces a robust inflammatory response, leading to increased cytokine expression in macrophages and human umbilical vein endothelial cells (HUVECs). These findings suggest that infosomes serve as a previously unrecognized mechanism of inflammasome-driven intercellular communication, amplifying immune responses beyond the initially activated immune cells.

The distinct molecular composition of infosomes suggests their potential utility as biomarkers for inflammatory diseases and as therapeutic targets for immune modulation. Given the increasing interest in targeting the NLRP3 inflammasome in various disease contexts, understanding the role of infosomes in inflammation may offer new insights into disease mechanisms and therapeutic strategies. By elucidating the composition and functional significance of inflammasome-associated EVs, this study provides a foundation for future research into the broader implications of inflammasome-regulated vesicular communication in disease pathology.

## Experimental Procedures

### Cell Culture and Treatments

THP-1 cells were purchased from Invivogen and cultured in complete medium containing Roswell Park Memorial Institute 1640 Medium (RPMI 1640 medium; Thermo Fisher Scientific, Inc., Gibco,; Cat. No. 11875) supplemented with 1% penicillin-streptomycin solution (Thermo Fisher Scientific, Inc.; Cat. No. 15240062) and 10% fetal bovine serum (FBS; Thermo Fisher Scientific, Inc.; Cat. No. 10099-141).

Isolation of bone marrow-derived macrophage (BMDMs) were conducted with the approval of the Animal Care and Use Committee of Gyeongsang National University (ACUC approval number: GNU-240906-M0180). BMDMs were isolated from 6-week-old male C57BL/6 mice. After euthanasia, femurs and tibias were harvested, and bone marrow was flushed out using a 26-gauge needle with Dulbecco's Modified Eagle Medium (DMEM; Thermo Fisher Scientific, Inc., Gibco; Cat. No. 11995065) supplemented with 1% penicillin-streptomycin and 10% FBS. The extracted bone marrow was centrifuged at 1500*g* for 5 min, and red blood cells were removed using red blood cells Lysis Buffer (Thermo Fisher Scientific, Inc., eBioscience, Cat. No. 00-4333-57). The pellet was resuspended in DMEM supplemented with 1% penicillin-streptomycin and 10% FBS, filtered through a 40 μm cell strainer (Corning Life Sciences, Corning; Cat. No. 431750), and cultured for 24 h. Monocytes were isolated from the supernatant (Sup), and the cells were differentiated in DMEM containing 1% penicillin-streptomycin, 10% FBS, and macrophage colony-stimulating factor (M-CSF; Thermo Fisher Scientific, Inc., PeproTech; Cat. No. 315-02) for 1 week before being used in experiments.

HUVECs were purchased from PromoCell (Heidelberg, Germany Cat. No. c-12203) and maintained in M199 (Medium 199; Sigma-Aldrich Co.; Cat. No. M4530) supplemented with 30 μg/ml endothelial cell growth supplement (Corning Life Sciences, Discovery labware; Cat. No. 356006), 100 μg/ml heparin (Sigma-Aldrich Co.; Cat. No. H3149) and 20% FBS (GenDEPOT; Cat. No. F0900-050).

All cells were cultured at 37 °C in a humidified atmosphere containing 5% CO_2_ using a Forma Steri-Cycle i160 CO_2_ Incubator (Thermo Fisher Scientific, Inc.; Cat. No. 51033554).

Lipopolysaccharides from *Escherichia* *coli* O111 (LPS; Cat. No. L2630) was purchased from Sigma-Aldrich. MCC950 (Cat. No. inh-mcc) and Nigericin (Cat. No. tlrl-nig) were purchased from InvivoGen. All reagents were stored and used according to the manufacturer's instructions.

### Extracellular Vesicles Isolation

THP-1 cells were seeded at a density of 5 × 10^6^ cells per well in a 6-well plate using RPMI 1640 medium supplemented with 1% penicillin-streptomycin, 10% FBS, and 10 nM phorbol 12-myristate 13-acetate (PMA; Sigma-Aldrich Co.; Cat. No. P8139). After 24 h of incubation to induce differentiation into macrophages, the medium was replaced with RPMI 1640 and without FBS. The cells were then primed with LPS (1 μg/ml) for 4 h and treated with nigericin (5 μM) for 1 h to activate the NLRP3 inflammasome. The supernatant was collected and centrifuged at 2000*g* for 30 min at 4 °C to remove cells. The resulting cell-free culture medium was mixed with total exosome isolation reagent (Thermo Fisher Scientific, Inc.; Cat. No. 4478359) at a 2:1 ratio. The solution was incubated overnight at 4 °C and then centrifuged at 10,000*g* for 1 h at 4 °C to precipitate the EVs. The supernatant was removed by aspiration, and the EVs at the bottom of the tube were resuspended in an appropriate buffer for subsequent analysis. Differentiated BMDMs were treated under the same conditions for NLRP3 inflammasome activation, and EVs were extracted using the same procedures.

### Protein Isolation and Measurement

Total protein from cells or EVs was extracted using M-PER Mammalian Protein Extraction Reagent (Thermo Fisher Scientific, Inc.; Cat. No. 78501) mixed with Xpert Duo Inhibitor Cocktail Solution (GenDEPOT; Cat. No. P3300-005). Protein concentration was measured using the Bicinchoninic acid protein assay kit (Thermo Fisher Scientific, Inc.; Cat. No. 23225) according to the manufacturer's instructions. The extracted protein concentrations were determined using the BioTek Cytation 7 Cell Imaging Multi-Mode Reader (BioTek Instruments Inc.) and analyzed with BioTek Gen5 (https://www.agilent.com/en/product/microplate-instrumentation/microplate-instrumentation-control-analysis-software/imager-reader-control-analysis-software/biotek-gen5-software-for-detection-1623227) software.

### Western Blot Analysis

Proteins extracted from cells were isolated using the M-PER Mammalian Protein Extraction Reagent along with the Xpert Duo Inhibitor Cocktail Solution. Protein concentration was measured using a bicinchoninic acid protein assay kit. Total protein extracted from whole cells was normalized across samples based on measured concentrations, while proteins isolated from EVs were analyzed based on the total amount extracted. Protein separation by Sodium Dodecyl Sulfate-Polyacrylamide Gel Electrophoresis (SDS-PAGE) and transfer were performed following the manufacturer's protocol using the Mini-PROTEAN Tetra Cell system (Bio-Rad Laboratories). Polyacrylamide gels were prepared using Acrylamide-Bis Solution 30% 29:1 (Bio-solution, Suwon, Korea; Cat. No. BA004a), 1.5 M Tris-Cl pH 8.8 with SDS (Bio-solution; Cat. No. BT024a), 1M Tris-HCl pH 6.8 (Bio-solution; Cat. No. BT015), 10% Ammonium Persulfate Solution (Bio-solution; Cat. No. BA013a), and 10% SDS Solution (Bio-solution; Cat. No. BS003a). Proteins separated by electrophoresis were transferred onto Immobilon-PSQ PVDF membranes (Merck Millipore, Cat. No. ISEQ00010) at 100 V for 2 h.

The PVDF membrane was blocked for 1 h in 2% bovine serum albumin (GenDEPOT; Cat. No. A0100-010) in Tris-buffered saline with Tween 20 (TBST; QuickSilver; Cat. No. EB1203), followed by three 15-min washes with TBST solution. Primary antibodies used for immunoblotting included High Mobility Group Box 1 (HMGB1) (Abcam Limited, Cambridge, UK; Cat. No. ab18256), copine-1 (Novusbio; Centennial, Cat. No. NBP1-32194), TUBA4a (Cell Signaling Technology, Inc.; Cat. No. #3878), Heat Shock Protein Family A Member 8 (HSPA8) (Santa Cruz Biotechnology Cat. No. SC-7298), TSG101 (Abcam Limited; Cat. No. ab125011), GAPDH (Cell Signaling Technology, Inc.; Cat. No. #2118), NLRP3 (AdipoGen Life Sciences Cat. No. AG-20B-0014-C100), IL-1β (Cell Signaling Technology, Inc.; Cat. No. #12703 or #12426), cleaved IL-1β (Cell Signaling Technology, Inc.; Cat. No. #83186 or #52718), Caspase-1 (Cell Signaling Technology, Inc.; Cat. No. #2225 or AdipoGen Life Sciences; Cat. No. AG-20B-0042–100), ASC (Santa Cruz Biotechnology, Cat. No. SC-514414 or Cell Signaling Technology, Inc.; Cat. No. #67824), and β-Actin (Cell Signaling Technology, Inc.; Cat. No. #3700). Horseradish peroxidase -conjugated anti-mouse (Cell Signaling Technology, Inc.; Cat. No. #7076) or anti-rabbit immunoglobulin G antibodies (Cell Signaling Technology, Inc.; Cat. No. #7074) was used as secondary antibodies.

Protein expression levels were detected using SuperSignal West Pico ECL (Thermo Fisher Scientific, Inc.; Cat. No. 34580) or SuperSignal West Femto Maximum Sensitivity Substrate (Thermo Fisher Scientific, Inc.; Cat. No. 34095). Detection and analysis were performed using the iBright 1500 system and iBright analysis (https://www.thermofisher.com/kr/ko/home/life-science/protein-biology/protein-assays-analysis/western-blotting/detect-proteins-western-blot/western-blot-imaging-analysis/ibright-systems/software.html) software (Thermo Fisher Scientific, Inc.).

### Acetone Precipitation of Proteins

Acetone precipitation was performed to extract proteins from the culture medium supernatant. The treated medium was centrifuged at 2000*g* for 30 min at 4 °C to remove cell debris. The supernatant was mixed with cold acetone at a 1:4 ratio and incubated overnight at −20 °C. The following day, the mixture was centrifuged at 15,000*g* for 15 min at 4 °C. The supernatant was carefully removed on ice without disturbing the pellet. The pellet was resuspended by vigorous vortexing in a solution of M-PER Mammalian Protein Extraction Reagent and Xpert Duo Inhibitor Cocktail Solution. To denature the proteins, 5X SDS-PAGE Loading Buffer containing 2-mercaptoethanol (Bio-solution; Cat. No. BS204) was added and gently vortexed. The mixture was then heated at 95 °C for 10 min. The extracted proteins were stored at −80 °C.

### ELISA

THP-1 cells were seeded at a density of 5 × 10^4^ cells/well in 96-well plates and were cultured with 10 nM PMA for 24 h to induce differentiation into macrophages. After replacing the medium with RPMI 1640 and without PMA, cells were primed with LPS (1 μg/ml) for 4 h and then activated with nigericin (5 μM) for 1 h. IL-1β secretion was measured using a Human IL-1 beta Uncoated ELISA Kit (Thermo Fisher Scientific, Inc.; Cat. No. 88-7261-77). The plates were analyzed with a BioTek Cytation 7 Cell Imaging Multi-Mode Reader and analyzed using BioTek Gen5 software.

### Nanoparticle Tracking Analysis

Nanoparticle tracking analysis was performed using the Zetaview instrument (Particle Metrix, Inning am Ammersee, Germany). The isolated EVs were resuspended in PBS filtered through a 0.1 μm filter. The dilution was adjusted to ensure that the detected particle count ranged between 150 and 200 particles. The sample was then transferred to a 5 ml syringe, carefully avoiding air bubbles, and injected into the Zetaview. All measurements were performed following the manufacturer's instructions.

### Coomassie Blue Staining

The isolated EVs were mixed with M-PER Mammalian Protein Extraction Reagent and Xpert Duo Inhibitor Cocktail Solution and vortexed to extract proteins from the EV. The extracted proteins were then mixed with 5X SDS-PAGE Loading Buffer containing 2-mercaptoethanol and heated at 95 °C for 10 min to denature the proteins. The EV protein samples were separated by electrophoresis on a 20% polyacrylamide gel.

The Coomassie blue staining solution was prepared by mixing 40% distilled water, 50% methanol (Merck Millipore; Cat. No. 67-56-1), 10% acetic acid (EMSURE acetic acid (glacial); Sigma-Aldrich, Supelco Cat. No. 1.00063.2511), and 0.1% Coomassie Brilliant Blue R-250 (Bio-Rad Laboratories; Cat. No. #161-0400). The destaining solution was prepared by mixing 50% distilled water, 40% methanol, and 10% acetic acid. Gel imaging was performed using the GelDoc Go (Bio-Rad Laboratories).

### Trypsin Digestion

Proteins extracted from EVs were separated by one-dimensional SDS-PAGE. Each gel lane was manually cut into equal-sized slices, yielding two sample fractions for control samples and four sample fractions for infosome samples, and all gel slices were further diced into 1 mm^3^ cubes using a scalpel. The gel slices were reduced for 45 min at 56 °C in 10 mM DTT/0.1 M ammonium bicarbonate and alkylated for 30 min at room temperature in the dark in 55 mM iodoacetamide/0.1 M ammonium bicarbonate. After washing with 50 mM ammonium bicarbonate/50% acetonitrile, gel cubes dried in a vacuum and were subjected to in-gel digestion using trypsin (Promega Cat. No. V5280). The resulting tryptic peptides were extracted in 50% acetonitrile/1% trifluoroacetic acid and purified by OASIS HLB 1 cc Vac Cartridge with 30 mg sorbent per cartridge (Waters Corporation, Milford Cat. No. WAT094225) prior to analysis by LC-MS/MS.

### Label-free Proteomics Analysis

Peptides were resuspended in 0.1% formic acid and separated on an Eksigent Ekspert nanoLC 415 system (SCIEX) coupled to a TripleTOF 6600 mass spectrometer (SCIEX, High-Tech Materials Core Facility at GNU). Following autosampler injection, samples were loaded onto a C18 NanoLC trap column (ChromXP, 3 μm, 120 Å; Part #5016752) at 2 μl/min for 10 min in 0.1% formic acid and eluted on a C18 analytical column (ChromXP, 3 μm, 120 Å; Part #805-00120) at 300 nl/min. The instrument was operated in information-dependent acquisition mode. Survey scans were acquired for 200 ms over 300-1800 m/z, and MS/MS spectra were collected on the top 20 precursors (charge 2–5; intensity >40 counts) over 80 to 2000 m/z with 150 ms accumulation time using rolling collision energy.

After MS/MS analysis, raw data were processed using SagePro (22) (v0.14.7; Chaparral Lab Inc.) implementing the mokapot framework and searched against the reviewed UniProt *Homo sapiens* proteome (20,420 entries; downloaded October 2025). Search parameters were: trypsin specificity with up to one missed cleavage; fixed carbamidomethylation of Cys; variable oxidation of Met; precursor and fragment ion tolerances set to 20 ppm. For label-free quantitative analysis, the Match-Between-Runs algorithm was enabled to improve quantitative completeness by propagating MS1-based quantitative values for peptides identified at the MS/MS level across runs. Peptide identifications were accepted at probabilities >95.0% corresponding to a global FDR <1.0% as estimated by the SagePro/mokapot workflow. Protein identifications were accepted at probabilities >99.0% with ≥2 unique peptides; as an additional stringency filter, ≥2 total and ≥2 unique spectral counts per protein were required. Significantly altered proteins were subjected to Kyoto Encyclopedia of Genes and Genomes, Gene Ontology, and miRNA target enrichment using ShinyGO (v0.85) (23). The proteomics dataset has been deposited to jPOSTrepo under JPST003860 (ProteomeXchange PXD064778).

### Experimental Design and Statistical Rationale

Label-free quantitative proteomics was performed on EVs from NLRP3 inflammasome-activated macrophages (LN) and non-stimulated macrophages (Control), each with three biological replicates. Control samples were fractionated into two fractions per biological replicate, whereas treatment samples were fractionated into four fractions. Each fraction was analyzed in two to three technical replicates. The mean intensity across technical replicates represented each fraction, and the summed intensity across fractions represented the corresponding biological replicate.

Within each experimental group, proteins missing in more than 70% of samples were excluded; proteins with 70% or fewer missing values in at least one group were retained, and missing values were imputed using the median of observed values. Intensities were log_2_-transformed and normalized for central tendency by aligning group medians to the sample with the highest median intensity. Differential abundance was assessed by fold change and Welch's *t* test; proteins with fold change ≥2 (log_2_ ≥ 1) and *p* < 0.05 were considered significantly altered. To ensure robustness, downstream analyses included only proteins meeting the identification criteria above (protein probability >99.0% with peptide and spectral count thresholds).

### RT-PCR Analysis

Total RNA was isolated using a NucleoSpin RNA (Macherey-Nagel, Düren, Nordrhein-Westfalen; Cat. No. 740955.250) according to the manufacturer's instructions. Collect the cells through scraping using cell scraper (SPL Life Sciences Co., Pocheon, Korea; Cat. No. 90020). To lysate the cells, add 10% 2-mercapotoethanol (BIOPURE, Cambridge Cat. No. 6230M) to 350 μl of buffer RA1 and transfer it on a violet ring column. Centrifuge 11,000*g* for 1 min and discard the column. Add 350 μl of 70% ethanol (Merck Cat. No. 1.00983.1011) to the lysate and gently pipetting. Transfer 700 μl lyse to blue ring column, centrifuge 11,000*g* for 30 s and discard the flowthrough. Add membrane desalting buffer buffer (350 μl), centrifuge 11,000*g* for 1 min and discard the flowthrough. Add DNase mixture (10 μl reconstituted rDNase plus 90 μl reaction buffer) and incubate for 15 min. Add RAW2 buffer (200 μl), and RA3 (600 μl or 250 μl) were sequentially added to the column, with centrifugation at 11,000*g* after each step, and the flow-through was discarded. Transfer to new collection tube and elute the RNA using 50 μl of RNase free water. Total RNA was reverse transcribed with iScript cDNA synthesis kit (Bio-Rad Laboratories; Cat. No. 1708891). 1 μg RNA, 4 μl of 5 X iScript reaction mix, 1 μl iScript reverse transcriptase and RNase free water were added into a 20 μl reverse transcriptase reaction. The reaction was completed under the following conditions: 25 °C for 5 min, 46 °C for 20 min, 95 °C for 1 min. Real-time PCR was performed using an iQ supermix SYBR Green (Bio-Rad Laboratories; Cat. No. BR1708884). cDNA and specific primer were added into the qPCR Mix and distilled water was added to a total volume of 10 μl. Real-time PCR was performed using a PCR thermal cycler (Thermo Fisher Scientific). Real-time PCR was performed using Rotor-Gene Q (https://www.qiagen.com/us/products/discovery-and-translational-research/epigenetics/dna-methylation/methylation-specific-pcr/rotor-gene-q?catno=9020147) and software v2.3.1 (QIAGEN N.V., Hilden, Germany). The used primers were described as follows the table. The RNA expression was calculated using 2^-△△Ct^ (Livak) Method.

**Table:** 

Name of Gene	Direction	Nucleotide Sequence
*IL-1α*	Forward	5′-TGTATGTGACTGCCCAAGATGAAG-3′
	Reverse	5′-AGAGGAGGTTGGTCTCACTACC-3′
*IL-1β*	Forward	5′-CCACAGACCTTCCAGGAGAATG-3′
	Reverse	5′-GTGCAGTTCAGTGATCGTACAGG-3′
*IL-6*	Forward	5′-AGACAGCCACTCACCTCTTCAG-3′
	Reverse	5′-TTCTGCCAGTGCCTCTTTGCTG-3′
*IL-8*	Forward	5′-GAGAGTGATTGAGAGTGGACCAC-3′
	Reverse	5′-CACAACCCTCTGCACCCAGTTT-3′
*MCP1*	Forward	5′-AGAATCACCAGCAGCAAGTGTCC-3′
	Reverse	5′-TCCTGAACCCACTTCTGCTTGG-3′
*MCP2*	Forward	5′-TATCCAGAGGCTGGAGAGCTAC-3′
	Reverse	5′-TGGAATCCCTGACCCATCTCTC-3′
*GM-CSF*	Forward	5′-GGAGCATGTGAATGCCATCCAG-3′
	Reverse	5′-CTGGAGGTCAAACATTTCTGAGAT-3′

### Statistical Analysis

All experimental data were visualized and ANOVA or two-tailed unpaired *t*-tests, as appropriate, in GraphPad Prism (https://www.graphpad.com/updates/prism-10-1-1-release-notes) software (Version 10; GraphPad Software Inc.). All statistical parameters, *p*-values, and other relevant detailed information are presented in figure legends.

## Results

### NLRP3 Inflammasome Activation Enhances Secretion of Infosomes Enriched with Proteome

**Fig. 1:**
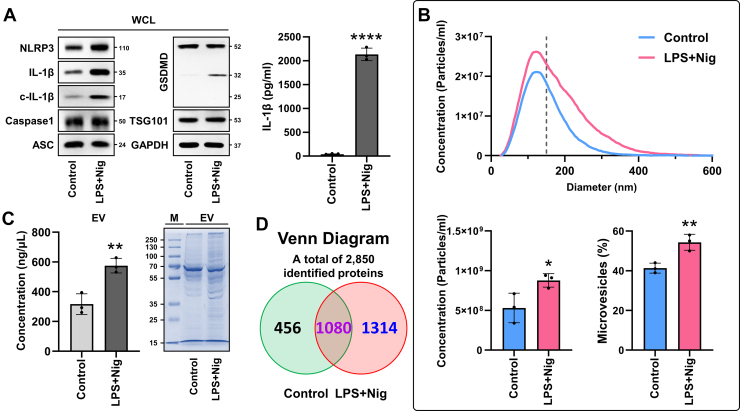
**NLR family pyrin domain-containing 3 (NLRP3) inflammasome activation increases the secretion of proteome-enriched infosomes.***A*, THP-1 cells were treated with LPS for 4 h followed by nigericin for 1 h. Protein expression levels in whole cell lysate (WCL) were analyzed by Western blot, and IL-1β secretion was quantified using ELISA. *B*, nanoparticle tracking analysis was used to measure the size and distribution of the isolated particles. The size distribution and total particle concentration per milliliter are shown. Percentage of particles within the 150 to 1000 nm range were defined as microvesicles. *C*, concentration of isolated EVs was measured, and their protein distribution was visualized using Coomassie staining. *D,* a Venn diagram showing the distribution of all proteins identified through qualitative proteomic analysis in conventional EVs and infosomes. Error bars show means ± SD with individual data points. Two-tailed unpaired *t* test analysis was conducted to determine statistical significance (∗*p* < 0.05, ∗∗*p* < 0.01, ∗∗∗*p* < 0.001, ∗∗∗∗*p* < 0.0001). WCL, marker, and EV is indicated in the figure. EV, extracellular vesicle; WCL, whole cell lysate; NLRP3, NLR family pyrin domain-containing 3.

**Figure S1:**
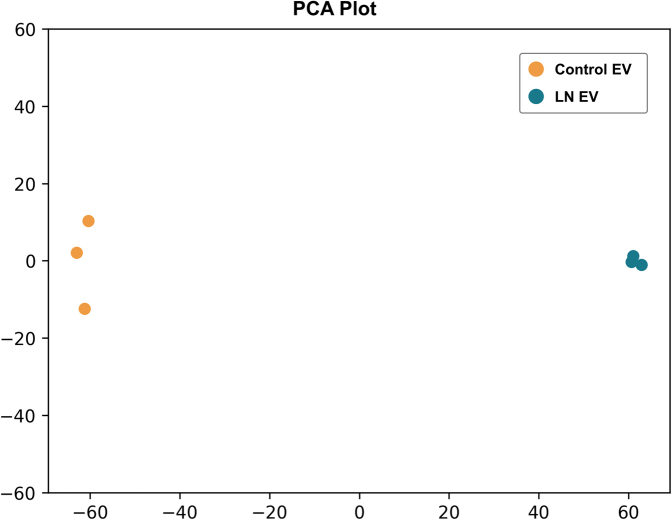


To investigate the relationship between NLRP3 inflammasome activation and EVs release, a model system was established to activate the NLRP3 inflammasome in macrophages (Fig. 1*A*). The activation of the inflammasome was confirmed by the increased expression of NLRP3 and IL-1β proteins, as well as the proteolytic maturation of IL-1β and gasdermin D (GSDMD). The significant increase in IL-1β secretion confirmed the successful establishment of the NLRP3 inflammasome activation model system. We investigated the characteristics of EVs released into the extracellular environment, including their particle size and number, and found that macrophages secreted particles ranging in size from 20 nm to 600 nm (Fig. 1*B*). In NLRP3 inflammasome-activated macrophages, the total number of secreted particles increased, with the size distribution shifting toward larger particles and a higher proportion of microvesicles (150–1000 nm). Similarly, we observed an increase in the concentration of proteins with various molecular weights within EVs secreted from NLRP3 inflammasome-activated macrophages, a characteristic termed infosomes (Fig. 1*C*). Therefore, we examined the protein composition and characteristics of infosomes through proteomic analysis (Supplemental Fig. S1). In the initial identification-based analysis, a total of 2850 proteins were identified across all samples by DDA-based MS/MS analysis. Among these, 1080 proteins were detected in both infosomes and conventional EVs, while 1314 proteins were uniquely detected in infosomes (Fig. 1*D*, Supplemental Table S1). These findings suggest that NLRP3 inflammasome activation alters the proteomic composition of infosomes, contributing to their functional heterogeneity.

### Enrichment of Distinct Proteomic Signatures and EV Marker Proteins in Infosomes

**Fig. 2:**
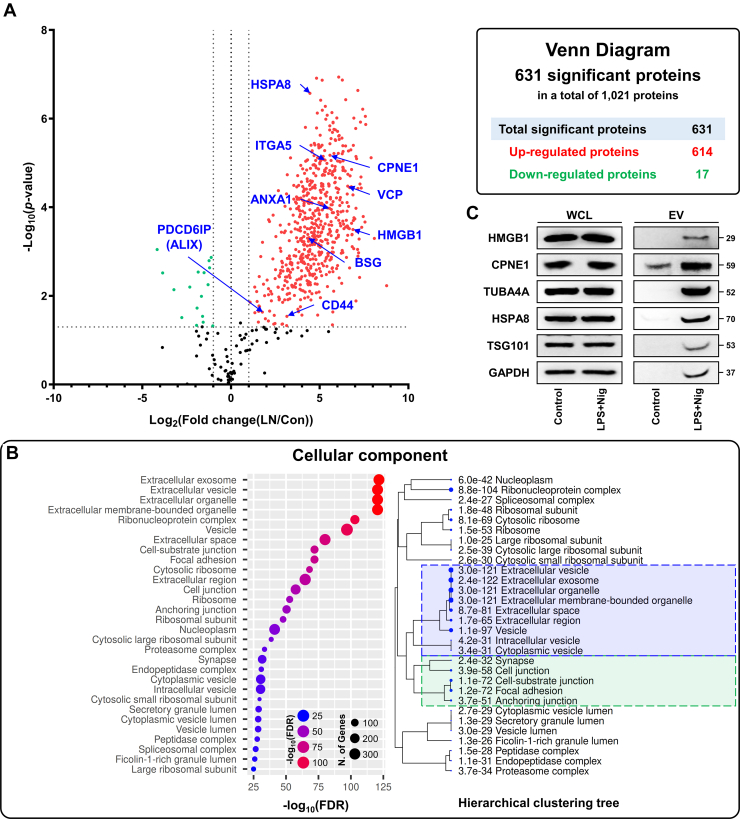
**Infosomes are enriched with EV marker proteins.***A*, volcano plot depicting the differential expression of proteins between infosomes (LN) and conventional EVs (Con). Proteins showing more than twofold upregulation in infosomes are marked in *red* (*n* = 614; *p*-value <0.05), while those with more than twofold downregulation are indicated in *green* (*n* = 17; *p*-value <0.05). The accompanying table summarizes the number of proteins showing significant increases or decreases in infosomes relative to conventional EVs. *B*, cellular component enrichment analysis of proteins upregulated in infosomes. *Green boxes* indicate junction- and adhesion-related compartments, and *blue boxes* indicate EV-related compartments. *C*, expression levels of EV-associated proteins, including High Mobility Group Box 1, copine-1, TUBA4A, Heat Shock Protein Family A Member 8, TSG101 and GAPDH were analyzed by Western blot. WCL and EVs are indicated in the figure. EV, extracellular vesicle; WCL, whole cell lysate.

From the quantitative analysis, a total of 1021 proteins were selected for downstream quantitative analysis based on the availability of reliable MS1-based quantitative values. To ensure analytical reliability, only proteins identified with at least two unique peptides were included. Among these, 631 proteins exhibited significant changes in abundance, defined by a fold change greater than 2 and a *p*-value less than 0.05 (Fig. 2*A*). Of the significantly altered proteins, 614 were increased in infosomes and 17 were decreased (Supplemental Table S2). Proteomic analysis further demonstrated that the upregulated proteins in infosomes were enriched in components generally recognized as canonical EV markers or known to be associated with EVs (24, 25). For instance, several EV-associated proteins, including Programmed cell death 6-interacting protein (PDCD6IP; ALIX), CD44, and basigin, were identified. Proteins involved in membrane dynamics and cellular interactions, such as valosin-containing protein and integrin α5, were also detected. In addition, immune- and stress-related proteins, including HSPA8, HMGB1, annexin A1, and copine-1, were present within infosomes. Similarly, cellular component enrichment analysis of the 631 upregulated proteins demonstrated a strong association with EV-related compartments, including extracellular exosomes, extracellular vesicles, membrane-bounded organelles, and vesicles (Fig. 2*B*, Supplemental Table S3). Consistent with the proteomic analysis, protein expression profiling further confirmed that the levels of representative canonical EV markers and EV-associated proteins were higher in infosomes than in conventional EVs (Fig. 2*C*). In addition, a significant enrichment of proteins associated with cell junctions such as cell-substrate junction, focal adhesion, and anchoring junction was observed. These findings not only reflect the enrichment of EV-associated proteins in infosomes but also indicate an altered protein composition under inflammatory conditions. The concordance between EV-related pathway enrichment and elevated marker expression supports both the reliability of the EV isolation process and the robustness of the proteomic profiling. Moreover, the presence of junctional proteins suggests a partial plasma membrane origin of infosomes and implies a potential role in modulating intercellular interactions.

### Infosome Proteins are Associated with Immune Pathways and Vesicle-Mediated Processes

**Fig. 3:**
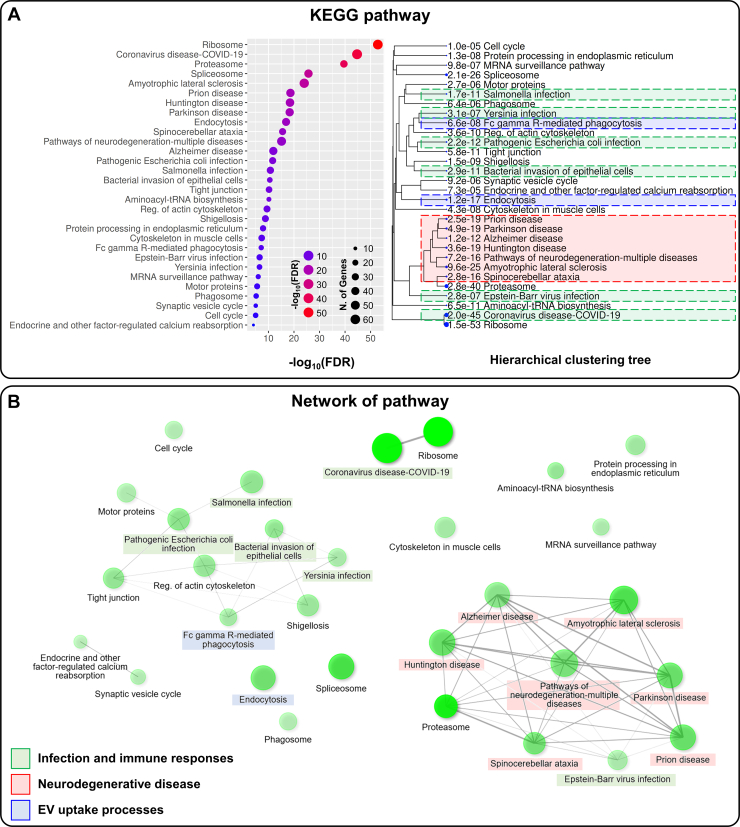
**Biological pathway analysis of infosomes.***A*, Kyoto Encyclopedia of Genes and Genome pathway enrichment analysis of proteins upregulated in infosomes. *Green boxes* indicate infection- and immune-related pathways, *blue boxes* represent EV uptake processes, and *red boxes* denote neurodegeneration-related pathways. *B*, network visualization of Kyoto Encyclopedia of Genes and Genome pathways enriched in proteins upregulated in infosomes. Functionally related pathways are clustered and color-coded as follows: *green* for infection and immune response, *red* for neurodegenerative diseases, and *blue* for EV uptake processes. EV, extracellular vesicle.

To elucidate the functional roles of proteins in infosomes, we performed a biological pathway analysis. Kyoto Encyclopedia of Genes and Genome analysis revealed that proteins enriched in infosomes were associated with biological pathways including infectious immune responses, neurodegenerative diseases, and extracellular vesicle uptake processes such as phagocytosis and endocytosis (Fig. 3*A*, Supplemental Table S4). Infection- and immune-related pathways (e.g., bacterial invasion of epithelial cells, *Salmonella* infection, pathogenic *Escherichia. coli* infection, COVID-19) were significantly enriched and commonly included components of the actin-related protein 2/3 (ARP2/3) complex. These pathways also featured major immune-associated proteins such as heat shock protein 90 kDa alpha family class A member 1 (HSP90AA1), HSP90 alpha class B member 1 (HSP90AB1), signal transducer and activator of transcription 1 (STAT1), and HMGB1.

Furthermore, a distinct cluster of neurodegeneration-related pathways was identified, including Alzheimer's disease, Parkinson's disease, amyotrophic lateral sclerosis, Huntington's disease, and prion disease (Fig. 3*B*). Frequently detected proteins in this cluster included tubulin isoforms, as well as proteasome 20S subunit beta, proteasome 26S subunit, ATPase, and proteasome 26S subunit, non-ATPase subunits. Pathways related to phagocytosis and endocytosis were also enriched, suggesting the presence of proteins involved in vesicle internalization within infosomes. Collectively, these findings highlight the distinct proteomic composition of infosomes and their potential roles in immune modulation, neurodegenerative disease mechanisms, and EV dynamics.

**Fig. 4:**
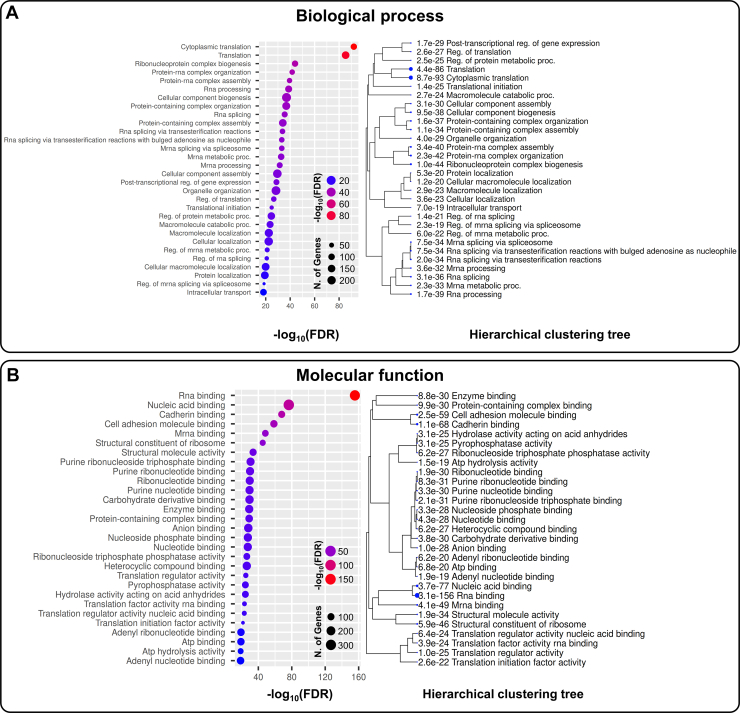
**Gene ontology terms enriched in proteins upregulated in infosomes.***A and B,* Gene ontology enrichment analysis of proteins upregulated in infosomes, with (*A*) Biological process and (*B*) Molecular function.

To further characterize the properties of infosomes, we examined the associated biological processes and molecular functions. Biological process analysis showed significant enrichment in pathways related to protein synthesis, translational regulation, ribosome-associated functions, and RNA metabolism, indicating that infosomes are rich in components important for gene expression and protein production (Fig. 4*A*, Supplemental Table S5). Molecular function analysis highlighted binding-related activities, including RNA-related binding, nucleic acid binding, and mRNA binding being particularly prominent (Fig. 4*B*, Supplemental Table S6). These observations suggest that infosome proteins may participate in regulating RNA stability or function.

### Infosomes are Generated in a Manner Dependent on NLRP3 Inflammasome Activation

**Fig. 5:**
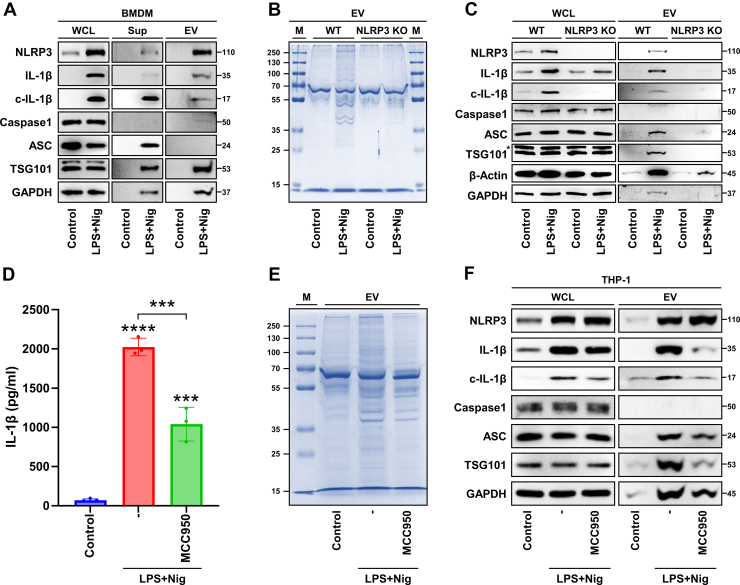
**Infosomes are secreted in an NLRP3 inflammasome-dependent manner.***A*, bone marrow-derived macrophages were primed with LPS for 4 h, followed by activation with nigericin for 1 h. Protein expression in WCLs, supernatants, and EVs was analyzed by Western blot. *B*, EV proteins from THP-1 WT and NLRP3-KO cells after NLRP3 inflammasome activation was analyzed by Coomassie staining. *C*, protein expression in WCLs and secreted EVs from THP-1 WT and NLRP3-KO cells was analyzed by Western blot. *D* and *F*, THP-1 cells were pretreated with MCC950 (NLRP3 inhibitor; 10 μM) for 30 min, followed by priming with LPS for 4 h and activation with nigericin for 1 h *D* IL-1β secretion was measured using ELISA. *E,* proteins isolated from EVs were analyzed by Coomassie staining. *F,* protein expression in WCLs and EVs was analyzed by Western blot. *Error bars* show means ± SD with individual data points. One-way ANOVA with Tukey's *post hoc* analysis was conducted to determine statistical significance (∗∗∗*p* < 0.001, ∗∗∗∗*p* < 0.0001; N.D., not detected). WCL, supernatant, EV, and marker (M) are indicated in the figure. EV, extracellular vesicle; WCL, whole cell lysate; NLRP3, NLR family pyrin domain-containing 3.

Previous studies reported that pyroptosis induced by NLRP3 inflammasome activation lead to the release of various intracellular molecules, including components of the NLRP3 inflammasome, which can be transferred to and influence recipient cells (26). EVs enriched with IL-1β were released via a GSDMD-dependent pathway activated by the NLRP3 inflammasome, and this process has been directly associated with the process of inflammatory bowel disease (27). Based on these findings, we hypothesized that upregulated components of the NLRP3 inflammasome are enriched within infosomes, despite their absence in the proteomic database due to low abundance. In BMDMs, we identified that NLRP3 inflammasome activation was accompanied by the proteolytic maturation of IL-1β and the extracellular release of NLRP3 and ASC (Fig. 5*A*). Under these conditions, consistent with previous findings, the increased expression of classical EV markers such as TSG101 confirmed the generation of infosomes. Notably, a substantial amount of NLRP3, along with IL-1β and its proteolytically matured form, was identified within infosomes, validating the enhanced generation of infosomes containing NLRP3 inflammasome components. Next, we hypothesized that the mechanism for infosome generation depends on the NLRP3 protein and the activation of the NLRP3 inflammasome. To investigate the role of NLRP3 in infosome production via the NLRP3 inflammasome pathway, the same inflammasome-activating stimuli were applied to NLRP3-KO macrophages, followed by isolation of EVs for comparative analysis. The elevated protein content within infosomes observed in macrophages was significantly reduced in NLRP3-KO macrophages (Fig. 5*B*). Furthermore, the expression levels of key proteins comprising the NLRP3 inflammasome were reduced to levels comparable to those in the control group (Fig. 5*C*). These findings suggest that the heterogeneous characteristics of infosomes are regulated by NLRP3 and that NLRP3 is a critical protein in the early stages of infosome generation. To confirm whether these changes are mediated by NLRP3 inflammasome activation, macrophages were treated with MCC950, an NLRP3 inhibitor that blocks ATP hydrolysis to inhibit inflammasome activation (28), and the results were compared. As predicted by our hypothesis, inhibition of NLRP3 inflammasome activation significantly reduced IL-1β secretion along with protein content of various molecular weights within infosomes (Fig. 5, *D* and *E*). Protein expression analysis demonstrated that when inflammasome activation was inhibited, the expression of NLRP3 inflammasome components within infosomes was also reduced (Fig. 5*F*). These findings provide further evidence that infosome generation is initiated by the NLRP3 protein and requires the activation of the NLRP3 inflammasome.

### Infosomes Induce Inflammation in Macrophages and Endothelial Cells

**Fig. 6:**
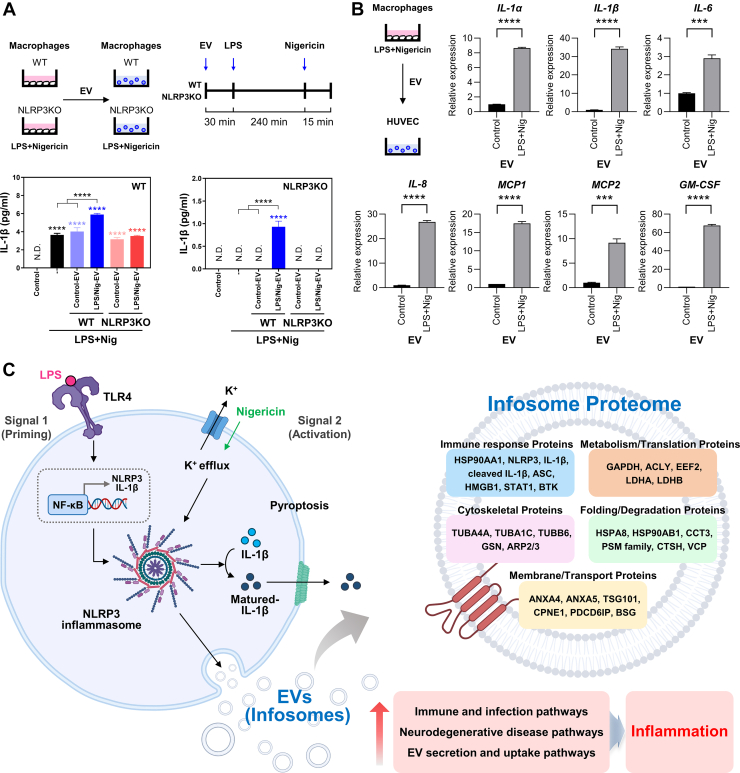
**Infosomes induce inflammatory responses in recipient cells.***A*, EVs isolated from macrophages were applied to macrophages, followed by inflammasome activation stimuli. IL-1β secretion was measured using ELISA. *B,* EVs derived from control and inflammasome-activated THP-1 cells were applied to HUVECs for 24 h to evaluate their effects on mRNA expression. mRNA expression levels were quantified using RT-PCR. *C*, schematic representation of proteomic profiling of EVs derived from macrophages with activated NLRP3 inflammasomes. The illustration was created using BioRender.com. Error bars show means ± SD. One-way ANOVA with Tukey's *post hoc* analysis and two-tailed unpaired *t* test analysis was conducted to determine statistical significance (∗∗∗*p* < 0.001, ∗∗∗∗*p* < 0.0001; N.D., not detected). EV, extracellular vesicle; NLRP3, NLR family pyrin domain-containing 3.

We confirmed that NLRP3 inflammasome activation enhanced infosome secretion and identified a strong association between infosome proteins and immune responses through proteomic analysis. Furthermore, infosomes were enriched with core components of the NLRP3 inflammasome, including NLRP3 and IL-1β, as well as immunoregulatory proteins such as heat-shock protein 90 family members (HSP90AA1, HSP90AB1) and HMGB1. Based on these findings, we hypothesized that infosomes, once transferred to recipient cells, can induce inflammation through various biological responses, including immune reactions. To investigate whether infosomes can induce immune responses in recipient cells, we evaluated infosomes secreted from WT and NLRP3-KO macrophages by applying them to WT and NLRP3-KO macrophages. In WT macrophages, a significant increase in IL-1β secretion was observed when infosomes derived from WT macrophages were co-treated with inflammasome activators, whereas infosomes derived from NLRP3-KO macrophages showed no significant effect (Fig. 6*A*). Similarly, IL-1β secretion was increased exclusively in NLRP3-KO macrophages treated with infosomes derived from WT macrophages, suggesting that infosomes have the potential to amplify inflammation. Next, to further investigate the ability of infosomes derived from immune cells to induce inflammation in other types of recipient cells, we examined their effects on HUVECs, a well-established model for studying vascular inflammation (29). Compared to infosomes derived from untreated macrophages, treatment with infosomes resulted in a significant upregulation of mRNA expression levels of pro-inflammatory genes, including IL-1β and interleukin 6 (IL-6), in HUVECs (Fig. 6*B*). These findings suggest that infosomes not only mediate inflammatory signaling between immune cells but also have the potential to influence the inflammatory state of non-immune recipient cells, underscoring their role as critical mediators of intercellular communication in inflammatory environments.

**Figure S2:**
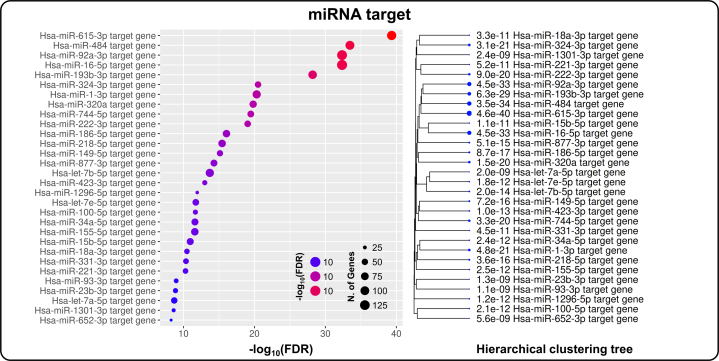


miRNAs, small non-coding RNA molecules that regulate genes by targeting messenger RNAs for degradation or translational repression, have been highlighted for their therapeutic potential in treating various diseases, including cancer, cardiovascular disorders, and viral infections (30). Building on our findings that infosomes are transferred to recipient cells and induce inflammation, we analyzed miRNAs predicted to target infosome-associated proteins to identify potential therapeutic candidates. As a result, we identified miR-615-3p, miR-16-5p, miR-92a-3p, miR-484, and miR-320 as key miRNAs capable of regulating infosome proteins (Supplemental Fig. S2, Supplemental Table S7). These miRNAs have the potential to suppress the expression of infosome proteins, thereby modulating biological processes in recipient cells affected by infosomes. This suggests that miRNA-based therapeutic strategies could be developed to mitigate the pathological effects of infosomes, providing a novel approach to treating inflammation-driven diseases.

## Discussion

Here, we provide evidence that NLRP3 inflammasome-dependent immune responses induce the generation of EVs, referred to as infosomes, which directly participate in amplifying inflammation (Fig. 6*C*). Our study confirmed that NLRP3 inflammasome activation significantly increased EV secretion from macrophages. This finding is consistent with a growing body of literature demonstrating that various inflammatory stimuli, including bacterial infection as well as both non-canonical and canonical inflammasome activation, enhance EV release and modulate their cargo (31, 32, 33). These studies established that inflammasome-activated EVs can carry protein or RNA cargos that influence recipient cells (33, 34). Our findings align with this precedent, supporting the reliability of our model. However, our work moves significantly beyond these observations to define the unique proteomic profile (identifying 614 upregulated and 17 downregulated proteins) and, more importantly, the specific functional role of these EVs.

The primary novelty of our study lies in demonstrating how these infosomes act as potent mediators of inflammation. We found they amplify existing inflammatory responses in homologous cells (macrophages) and induce de novo inflammation in both heterologous cells (HUVECs) and inflammation-abrogated homologous cells. Crucially, we established that this pro-inflammatory function is dependent on the NLRP3 inflammasome, as EVs derived from NLRP3-KO macrophages failed to transmit this inflammatory signal, highlighting a specific, NLRP3-dependent mechanism of cell-to-cell immune communication.

HMGB1, which is abundant in the infosome fraction, is released during necrosis or immune activation and functions as a DAMP that promotes the production of IL-1β, IL-6, and TNF-α. EVs from LPS-stimulated macrophages reportedly carry intracellular HMGB1, contributing to hepatocyte pyroptosis and astrocyte inflammation via NF-κB activation (18, 35). In line with these findings, our results suggest that the enhanced expression of IL-1β and IL-6 observed in HUVECs treated with infosomes may be mediated by the delivery of HMGB1. Further studies are warranted to elucidate the underlying mechanisms and to explore the therapeutic potential of HMGB1 in EV-associated inflammation. In addition to HMGB1, we identified a broader set of immune-related cargos, such as STAT1, BTK, PARP1, and Ribosomal Protein S3. STAT1 acts as a key effector of interferon signaling, while PARP1 enhances inflammatory responses by increasing ROS production (36). BTK plays a key role in inflammatory signaling by mediating the activation of NF-κB and MAPK pathways downstream of immune receptor engagement (37). Ribosomal Protein S3 has been reported to induce proinflammatory cytokines such as IL-1β, IL-6, and TNF-α (38, 39). Together with heat shock proteins (HSP90AA1, HSP90AB1, and HSPA8), which stabilize innate immune signaling complexes, these proteins represent plausible mediators of EV-driven immune communication. Although the direct inflammatory effects of EV-delivered immune-related proteins require further validation, their overall enrichment supports the hypothesis that infosomes may function as mediators of inflammatory signaling in recipient cells. In addition to these molecules, our dataset included several immune-associated proteins such as ROCK1, ARHGEF1, ARHGEF2, HCLS1, and nucleolin. These factors contribute to pathways involved in innate and adaptive immune signaling, cytoskeletal organization, and inflammatory activation, suggesting that infosomes comprise an expanded spectrum of immune-relevant cargos capable of modulating recipient-cell responses (40, 41, 42, 43, 44, 45, 46).

We identified HSPA8 protein within infosomes associated with neurodegeneration. A previous study demonstrated that HSPA8 expression was increased in a spinal cord ischemia-reperfusion injury model, and its inhibition suppressed the expression and activation of the NLRP3 inflammasome, ultimately improving spinal cord tissue damage and promoting motor function recovery (47). Since EVs can cross various biological barriers, including the blood-brain barrier, through intercellular and transcellular pathways (48), our findings suggest that infosomes, including HSPA8, secreted by NLRP3 inflammasome activation, may cross the blood-brain barrier and contribute to neurodegenerative diseases. Moreover, considering that NLRP3 inflammasome-activated microglia, the primary immune cells in the brain, play a crucial role in neurodegeneration through DAMPs released from damaged or dying neurons (49), our results in macrophages similarly suggest that infosomes may contribute to neurodegenerative diseases. Therefore, investigating the characteristics and mechanisms of infosomes derived from immune responses in both brain and peripheral tissues will be essential for understanding disease pathology.

In our study, miR-615-3p and miR-16-5p were identified as the top miRNAs capable of targeting proteins validated to be enriched in infosomes. A previous study demonstrated that hypoxia downregulated miR-615-3p expression in human cardiomyocytes, whereas its overexpression reduced apoptosis and oxidative stress, suggesting that miR-615-3p may play a crucial role in suppressing cell death and reactive oxygen species production (50). In the caloric restriction model, the expression of miR-16-5p increased in the spleen, thymus, and colon, which suppressed the mRNA expression of IL-1β, IL-6, and TNF-α during LPS stimulation, suggesting that miR-16-5p may be an important factor involved in anti-inflammatory effects (51). Our findings suggest that miR-615-3p and miR-16-5p therapeutics may reduce the expression of proteins, such as HSP90AB1 and HSP90AA1, which can induce immune and inflammatory responses in progenitor cells. This may inhibit their enrichment in infosomes, thereby suppressing immune and inflammatory responses in recipient cells. Further studies are required to elucidate the underlying mechanisms and functions of miRNAs in infosomes.

In this study, we demonstrated the enrichment of infosomes with proteins associated with immune responses and degenerative brain diseases. Infosomes promoted the expression of inflammatory markers in non-immune cells, such as HUVECs. Our findings suggest that infosomes secreted from NLRP3 inflammasome-activated cells may not only directly contribute to disease progression in inflammatory conditions but also be transported to various organs, thereby playing a role in tissue damage. In relation to the results, future studies are needed to investigate the relationship between the generation of infosomes and the activation of NLRP3 inflammasomes, focusing on caspase-1 and ASC, which provide proteolytic enzymes and structural assembly. In addition, future studies are needed to explore the connection between infosomes and the mechanisms of the endosomal sorting complex required for transport pathway, as well as their correlation with disease. Several studies have reported that exosomes released from the endosomal system increase in cells undergoing cell death, such as apoptosis, necroptosis, and pyroptosis, and play a significant role in diseases including inflammation, cancer, and autoimmune disorders (52). During necroptosis and pyroptosis, cell membrane rupture mediated by mixed lineage kinase domain-like protein and GSDMD facilitated the release of EVs, which was associated with IL-1β release and inflammatory bowel disease (27). Therefore, further studies are required to investigate the generation and secretion of infosomes induced by cell membrane rupture, such as that mediated by GSDMD, in relation to disease onset and progression.

**Figure S3:**
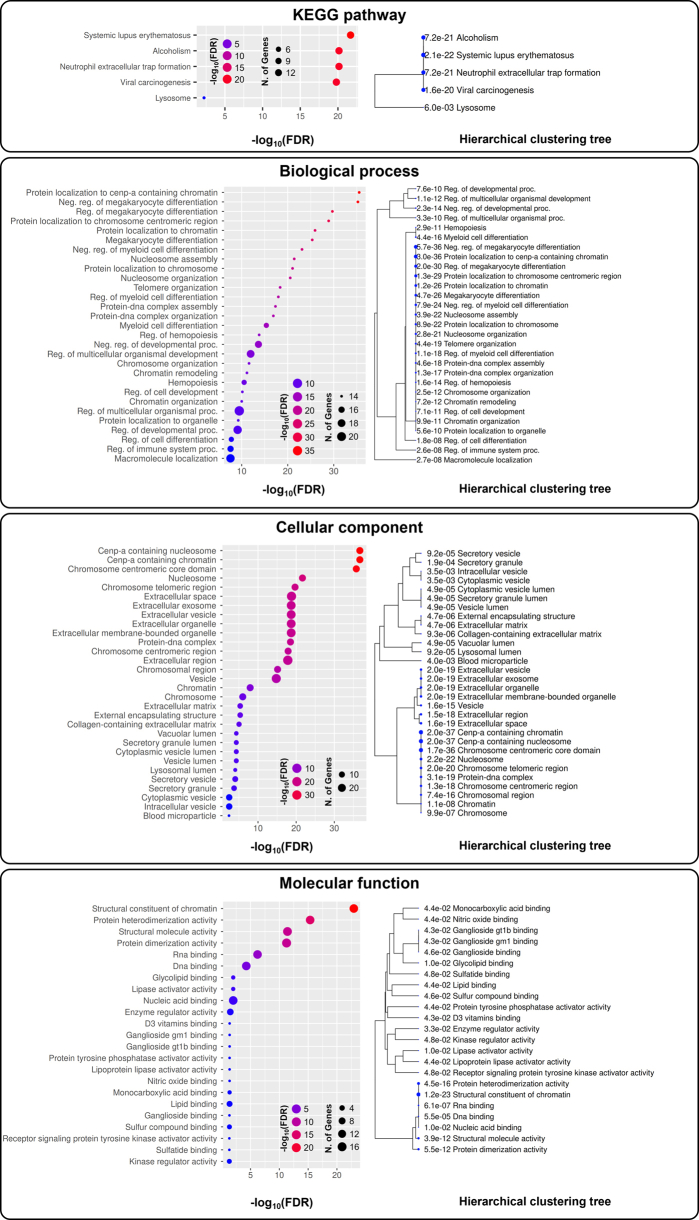


In addition to identifying proteins enriched in infosomes, proteomic profiling also revealed a subset of proteins whose abundance was significantly reduced in EVs derived from NLRP3 inflammasome-activated macrophages (Supplemental Fig. S3, Supplemental Table S8). Notably, lysosome-related proteins were among the underrepresented components. Although the mechanism underlying this selective reduction remains unclear, one possible explanation is that the preferential incorporation of inflammatory or signaling proteins during vesicle formation may competitively limit the inclusion of lysosomal components. Furthermore, inflammatory conditions may alter endosomal trafficking or EV biogenesis pathways in ways that reduce the sorting of lysosome-derived proteins into EVs.

Despite the strengths of *in vitro* studies, several limitations of our work should be considered. In our study, we used the immortalized macrophage cell line, including WT and NLRP3-KO THP-1 cells, to investigate the mechanisms of infosomes and validate their proteomic profiles and biological function, which exhibit an infosome phenotype. Although cross-validation was performed using primary macrophages, specifically BMDMs, further studies employing primary macrophages derived from NLRP3-KO mice will be required to elucidate the precise mechanisms and biological functions of infosomes.

Another potential limitation of our study is that, while we successfully analyzed a large quantity of proteins for the proteomic profiling of infosomes, the inherent sensitivity constraints of the MS-based proteomics methodology and/or the specific MS instrumentation used may have prevented the consistent detection of low-abundance proteins. This sensitivity challenge is particularly relevant when profiling key regulatory components, which are often present at lower cellular concentrations compared to abundant proteins, such as cytoskeletal proteins. In line with this, key inflammasome components, including NLRP3 and IL-1β, were clearly and readily detected by Western blot, but were not consistently identified in the proteomic dataset. This discrepancy suggests that these specific, low-concentration proteins existed below the Limit of Detection of the MS platform used.

Beyond the protein analysis, it should be considered that the function of infosomes may be influenced by a complex regulatory network mediated not only by proteins delivered to recipient cells but also by a substantial amount of miRNAs. The overall biological impact of the infosome and its corresponding EVs extends beyond the identified proteome, involving a dynamic interplay between protein effectors and non-coding RNA regulators. Therefore, an integrated analysis of both the proteome and the transcriptome of the infosome and its associated EVs is essential. Such a comprehensive approach is required to fully elucidate the complete molecular mechanism of infosome-mediated communication, providing a holistic view of the signaling pathways governed by both the protein machinery and their corresponding nucleic acid regulators.

To conclude, our findings suggest that EVs derived from macrophages in an NLRP3 inflammasome-activated immune response possess a unique proteomic profile. Furthermore, we identified novel EV proteins in NLRP3 inflammasome-activated macrophages that were not observed in other types of EVs, thereby expanding our understanding of macrophage-derived EV proteomes. In addition, our findings propose a novel concept that immune responses induce EVs containing various intracellular inflammatory factors, which directly contribute to inflammation. This concept provides new insights into the importance of developing therapeutics targeting infosomes for the treatment of inflammatory and neurodegenerative diseases.

## Data Availability

The mass spectrometry proteomics data generated in this study have been deposited in the jPOST repository (Japan ProteOme STandard Repository) under the accession number JPST003860 and are also available via the ProteomeXchange Consortium with the identifier PXD064778.

## Supplemental Data

This article contains supplemental data.

## Conflicts of Interest

The authors declare no conflict of interest.

## References

[1] Buzas E.I. (2023). The roles of extracellular vesicles in the immune system. Nat. Rev. Immunol..

[2] Crewe C. (2023). Energetic stress-induced metabolic regulation by extracellular vesicles. Compr. Physiol..

[3] Takasugi M. (2018). Emerging roles of extracellular vesicles in cellular senescence and aging. Aging Cell.

[4] van Niel G., D'Angelo G., Raposo G. (2018). Shedding light on the cell biology of extracellular vesicles. Nat. Rev. Mol. Cell Biol..

[5] Lombardi M., Parolisi R., Scaroni F., Bonfanti E., Gualerzi A., Gabrielli M. (2019). Detrimental and protective action of microglial extracellular vesicles on myelin lesions: astrocyte involvement in remyelination failure. Acta Neuropathol..

[6] Gong T., Liu L., Jiang W., Zhou R. (2020). DAMP-sensing receptors in sterile inflammation and inflammatory diseases. Nat. Rev. Immunol..

[7] Li D., Wu M. (2021). Pattern recognition receptors in health and diseases. Signal. Transduct. Target. Ther..

[8] Vande Walle L., Lamkanfi M. (2024). Drugging the NLRP3 inflammasome: from signalling mechanisms to therapeutic targets. Nat. Rev. Drug Discov..

[9] Swanson K.V., Deng M., Ting J.P. (2019). The NLRP3 inflammasome: molecular activation and regulation to therapeutics. Nat. Rev. Immunol..

[10] Yang Y., Wang H., Kouadir M., Song H., Shi F. (2019). Recent advances in the mechanisms of NLRP3 inflammasome activation and its inhibitors. Cell Death Dis..

[11] Devant P., Kagan J.C. (2023). Molecular mechanisms of gasdermin D pore-forming activity. Nat. Immunol..

[12] Kwon H.K., Lee I., Yu K.E., Cahill S.V., Alder K.D., Lee S. (2021). Dual therapeutic targeting of intra-articular inflammation and intracellular bacteria enhances chondroprotection in septic arthritis. Sci. Adv..

[13] Kwon H.K., Yu K.E., Cahill S.V., Alder K.D., Dussik C.M., Kim S.H. (2022). Concurrent targeting of glycolysis in bacteria and host cell inflammation in septic arthritis. EMBO Mol. Med..

[14] Kwon H.K., Dussik C.M., Kim S.H., Kyriakides T.R., Oh I., Lee F.Y. (2022). Treating “Septic” with enhanced antibiotics and “Arthritis” by mitigation of excessive inflammation. Front. Cell Infect. Microbiol..

[15] Jeljeli M.M., Adamopoulos I.E. (2023). Innate immune memory in inflammatory arthritis. Nat. Rev. Rheumatol..

[16] Ramachandran R., Manan A., Kim J., Choi S. (2024). NLRP3 inflammasome: a key player in the pathogenesis of life-style disorders. Exp. Mol. Med..

[17] Voet S., Srinivasan S., Lamkanfi M., van Loo G. (2019). Inflammasomes in neuroinflammatory and neurodegenerative diseases. EMBO Mol. Med..

[18] Wang G., Jin S., Huang W., Li Y., Wang J., Ling X. (2021). LPS-induced macrophage HMGB1-loaded extracellular vesicles trigger hepatocyte pyroptosis by activating the NLRP3 inflammasome. Cell Death Discov..

[19] Hosseinkhani B., van den Akker N.M.S., Molin D.G.M., Michiels L. (2020). (Sub)populations of extracellular vesicles released by TNF-alpha -triggered human endothelial cells promote vascular inflammation and monocyte migration. J. Extracell Vesicles.

[20] Tang N., Sun B., Gupta A., Rempel H., Pulliam L. (2016). Monocyte exosomes induce adhesion molecules and cytokines via activation of NF-kappaB in endothelial cells. FASEB J..

[21] Buzas E.I., Gyorgy B., Nagy G., Falus A., Gay S. (2014). Emerging role of extracellular vesicles in inflammatory diseases. Nat. Rev. Rheumatol..

[22] Lazear M.R. (2023). Sage: an open-source tool for fast proteomics searching and quantification at scale. J. Proteome Res..

[23] Ge S.X., Jung D., Yao R. (2020). ShinyGO: a graphical gene-set enrichment tool for animals and plants. Bioinformatics.

[24] Asleh K., Dery V., Taylor C., Davey M., Djeungoue-Petga M.A., Ouellette R.J. (2023). Extracellular vesicle-based liquid biopsy biomarkers and their application in precision immuno-oncology. Biomark Res..

[25] Chitti S.V., Gummadi S., Kang T., Shahi S., Marzan A.L., Nedeva C. (2024). Vesiclepedia 2024: an extracellular vesicles and extracellular particles repository. Nucleic Acids Res..

[26] Baroja-Mazo A., Martin-Sanchez F., Gomez A.I., Martinez C.M., Amores-Iniesta J., Compan V. (2014). The NLRP3 inflammasome is released as a particulate danger signal that amplifies the inflammatory response. Nat. Immunol..

[27] Bulek K., Zhao J., Liao Y., Rana N., Corridoni D., Antanaviciute A. (2020). Epithelial-derived gasdermin D mediates nonlytic IL-1beta release during experimental colitis. J. Clin. Invest..

[28] Li H., Guan Y., Liang B., Ding P., Hou X., Wei W. (2022). Therapeutic potential of MCC950, a specific inhibitor of NLRP3 inflammasome. Eur. J. Pharmacol..

[29] Mako V., Czucz J., Weiszhar Z., Herczenik E., Matko J., Prohaszka Z. (2010). Proinflammatory activation pattern of human umbilical vein endothelial cells induced by IL-1beta, TNF-alpha, and LPS. Cytometry A.

[30] Rupaimoole R., Slack F.J. (2017). MicroRNA therapeutics: towards a new era for the management of cancer and other diseases. Nat. Rev. Drug Discov..

[31] Lim Y., Kim H.Y., Han D., Choi B.K. (2023). Proteome and immune responses of extracellular vesicles derived from macrophages infected with the periodontal pathogen Tannerella forsythia. J. Extracell Vesicles.

[32] Cypryk W., Czernek L., Horodecka K., Chrzanowski J., Stanczak M., Nurmi K. (2023). Lipopolysaccharide primes human macrophages for noncanonical inflammasome-induced extracellular vesicle secretion. J. Immunol..

[33] Zhang Y., Liu F., Yuan Y., Jin C., Chang C., Zhu Y. (2017). Inflammasome-derived exosomes activate NF-kappaB signaling in macrophages. J. Proteome Res..

[34] Budden C.F., Gearing L.J., Kaiser R., Standke L., Hertzog P.J., Latz E. (2021). Inflammasome-induced extracellular vesicles harbour distinct RNA signatures and alter bystander macrophage responses. J. Extracell Vesicles.

[35] Kaya Z., Belder N., Sever-Bahcekapili M., Donmez-Demir B., Erdener S.E., Bozbeyoglu N. (2023). Vesicular HMGB1 release from neurons stressed with spreading depolarization enables confined inflammatory signaling to astrocytes. J. Neuroinflam..

[36] Chiu L.Y., Huang D.Y., Lin W.W. (2022). PARP-1 regulates inflammasome activity by poly-ADP-ribosylation of NLRP3 and interaction with TXNIP in primary macrophages. Cell Mol. Life Sci..

[37] Neys S.F.H., Hendriks R.W., Corneth O.B.J. (2021). Targeting Bruton's tyrosine kinase in inflammatory and autoimmune pathologies. Front Cell Dev Biol..

[38] Dong J., Liao W., Peh H.Y., Tan W.S.D., Zhou S., Wong W.S.F. (2018). Ribosomal protein S3 gene silencing protects against cigarette smoke-induced acute lung injury. Mol. Ther. Nucleic Acids.

[39] Zhao D., Zhang L., Song M., Mail Y.Z. (2022). RPS3-induced antiviral cytokines inhibit the proliferation of classical swine fever virus. Acta Virol..

[40] Gong J., Guan L., Tian P., Li C., Zhang Y. (2018). Rho kinase type 1 (ROCK1) promotes lipopolysaccharide-induced inflammation in corneal epithelial cells by activating toll-like receptor 4 (TLR4)-mediated signaling. Med. Sci. Monit..

[41] Glotfelty E.J., Tovar Y.R.L.B., Hsueh S.C., Tweedie D., Li Y., Harvey B.K. (2023). The RhoA-ROCK1/ROCK2 pathway exacerbates inflammatory signaling in immortalized and primary microglia. Cells.

[42] Chiang H.S., Zhao Y., Song J.H., Liu S., Wang N., Terhorst C. (2014). GEF-H1 controls microtubule-dependent sensing of nucleic acids for antiviral host defenses. Nat. Immunol..

[43] Brown J.P., Taube C., Miyahara N., Koya T., Pelanda R., Gelfand E.W. (2007). Arhgef1 is required by T cells for the development of airway hyperreactivity and inflammation. Am. J. Respir. Crit. Care Med..

[44] Gomez T.S., McCarney S.D., Carrizosa E., Labno C.M., Comiskey E.O., Nolz J.C. (2006). HS1 functions as an essential actin-regulatory adaptor protein at the immune synapse. Immunity.

[45] Kahner B.N., Dorsam R.T., Kim S., Shankar H., Kitamura D., Kunapuli S.P. (2008). Hematopoietic lineage cell-specific protein-1 (HS1) regulates PAR-mediated ERK activation and thromboxane generation in platelets. Platelets.

[46] Kitagawa S., Matsuda T., Washizaki A., Murakami H., Yamamoto T., Yoshioka Y. (2022). Elucidation of the role of nucleolin as a cell surface receptor for nucleic acid-based adjuvants. NPJ Vaccin..

[47] Mi J., Yang Y., Yao H., Huan Z., Xu C., Ren Z. (2021). Inhibition of heat shock protein family A member 8 attenuates spinal cord ischemia-reperfusion injury via astrocyte NF-kappaB/NLRP3 inflammasome pathway : HSPA8 inhibition protects spinal ischemia-reperfusion injury. J. Neuroinflam..

[48] Matsumoto J., Stewart T., Sheng L., Li N., Bullock K., Song N. (2017). Transmission of alpha-synuclein-containing erythrocyte-derived extracellular vesicles across the blood-brain barrier via adsorptive mediated transcytosis: another mechanism for initiation and progression of Parkinson's disease?. Acta Neuropathol. Commun..

[49] Heneka M.T., McManus R.M., Latz E. (2018). Inflammasome signalling in brain function and neurodegenerative disease. Nat. Rev. Neurosci..

[50] Zhang D., Zhang G., Yu K., Zhang X., Jiang A. (2022). MiRNA-615-3p alleviates oxidative stress injury of human cardiomyocytes via PI3K/Akt signaling by targeting MEF2A. Anatol J. Cardiol..

[51] Yamada K., Takizawa S., Ohgaku Y., Asami T., Furuya K., Yamamoto K. (2020). MicroRNA 16-5p is upregulated in calorie-restricted mice and modulates inflammatory cytokines of macrophages. Gene.

[52] Xiong M., Chen Z., Tian J., Peng Y., Song D., Zhang L. (2024). Exosomes derived from programmed cell death: mechanism and biological significance. Cell Commun. Signal..

