# Supplementary material for: Infosomes as Inflammatory Mediators: Proteomic Profiling of Proteins Enriched in Inflammatory Extracellular Vesicles
Source: Mol Cell Proteomics. 2026 Jan 19;25(2):101511. doi: 10.1016/j.mcpro.2026.101511 (PMC12925278; doi:10.1016/j.mcpro.2026.101511)
Supplement: Supplemental Data [file mmc9.docx]

**Supplemental Fig. S1** **Principal component analysis (PCA) of proteomic profiles.** The PCA plot shows distinct clustering of infosome samples (LN EV) compared with conventional EVs (Control EV), indicating global differences in their proteomic compositions. PC1 and PC2 represent the major axes of variance among the samples. PCA was performed using MS1 intensity values from three independently conducted biological replicates of infosomes and conventional EVs.

**Supplemental Fig. S2 Analysis of miRNAs targeting proteins within infosomes.** miRNA target gene enrichment analysis was performed using ShinyGO on 614 proteins significantly increased in infosomes compared to conventional EVs. The left panel displays the top enriched miRNAs whose validated target genes significantly overlapped with the infosome-enriched protein dataset. Dot color indicates the -log_10_(FDR) of enrichment, and dot size represents the number of mapped target genes from the dataset. The right panel shows a hierarchical clustering of the enriched miRNAs based on gene set similarity.

**Supplemental Fig. S3 KEGG and GO enrichment analysis of proteins significantly decreased in infosomes.** KEGG pathway and Gene Ontology (GO) enrichment analyses were performed using ShinyGO (v0.85) on 17 proteins that were significantly downregulated in infosomes compared to EVs from non-stimulated macrophages. Enriched terms include biological processes, cellular components, and molecular functions. Dot color indicates the -log_10_(FDR) of enrichment, and dot size represents the number of mapped target genes from the dataset. The right panel shows a hierarchical clustering of the enriched miRNAs based on gene set similarity.

**Supplemental Table. 1 Total identified protein.** This table provides a comprehensive list of all proteins identified by LC-MS/MS analysis, along with percent sequence coverage, the number of unique peptides, and MS1 intensity obtained from three biological replicates of conventional EVs (Control #1-3) and infosomes (LN #1-3).

**Supplemental Table. 2 Information on 631 significantly changed proteins.** This table presents 631 proteins that were significantly altered in infosomes compared with conventional EVs, based on the criteria of at least a two-fold change and *P* < 0.05. The table includes protein ID, protein name, gene symbol, protein description, sequence coverage, the number of unique peptides, MS1 intensities from three biological replicates (LN#1-3 and Cont#1-3), median values, log_2_ fold change, and *P*-values.

**Supplemental Table. 3 Cellular component enrichment analysis of proteins significantly upregulated in infosomes.** This supplemental table presents the results of Gene Ontology (GO) cellular component enrichment analysis, performed using ShinyGO (v0.85) on 614 proteins that were significantly increased in infosomes compared to conventional EVs. The table includes along with the enrichment false discovery rate (FDR), number of mapped genes from the dataset (nGenes), total number of genes annotated to each pathway (Pathway Genes), fold enrichment, and pathway name with its associated URL. The listed genes represent those from our dataset that contributed to each pathway. This dataset serves as the source data for Figure 2B.

**Supplemental Table. 4 KEGG enrichment analysis of proteins significantly upregulated in infosomes.** This supplemental table presents the results of Kyoto Encyclopedia of Genes and Genomes (KEGG) pathway enrichment analysis, performed using ShinyGO (v0.85) on 614 proteins that were significantly increased in infosomes compared to conventional EVs. The table includes along with the enrichment false discovery rate (FDR), number of mapped genes from the dataset (nGenes), total number of genes annotated to each pathway (Pathway Genes), fold enrichment, and pathway name with its associated URL. The listed genes represent those from our dataset that contributed to each pathway. This dataset serves as the source data for Figure 3A.

**Supplemental Table. 5 Biological process enrichment analysis of proteins significantly upregulated in infosomes.** This supplementary table presents the results of Gene Ontology (GO) biological process enrichment analysis, performed using ShinyGO (v0.85) on 614 proteins that were significantly increased in infosomes compared to conventional EVs. The table includes along with the enrichment false discovery rate (FDR), number of mapped genes from the dataset (nGenes), total number of genes annotated to each pathway (Pathway Genes), fold enrichment, and pathway name with its associated URL. The listed genes represent those from our dataset that contributed to each pathway. This dataset serves as the source data for Figure 4A.

**Supplemental Table. 6 Molecular function enrichment analysis of proteins significantly upregulated in infosomes.** This supplementary table presents the results of Gene Ontology (GO) molecular function enrichment analysis, performed using ShinyGO (v0.85) on 614 proteins that were significantly increased in infosomes compared to conventional EVs. The table includes along with the enrichment false discovery rate (FDR), number of mapped genes from the dataset (nGenes), total number of genes annotated to each pathway (Pathway Genes), fold enrichment, and pathway name with its associated URL. The listed genes represent those from our dataset that contributed to each pathway. This dataset serves as the source data for Figure 4B.

**Supplemental Table. 7 Enriched microRNAs predicted to regulate proteins significantly upregulated in infosomes.** This table summarizes the results of miRNA target enrichment analysis using ShinyGO (v0.85), aimed at identifying microRNAs predicted to regulate the proteins significantly increased in infosomes. Enrichment FDR indicates the adjusted false discovery rate (FDR) for each enriched miRNA pathway. nGenes denotes the number of input genes mapped to each miRNA target pathway. Pathway Genes refers to the total number of genes in the corresponding miRNA target gene set. Fold Enrichment shows the degree to which the input gene set is enriched in each miRNA pathway. Pathway lists the name of each enriched miRNA pathway. URL provides the reference link to the corresponding entry in the database. The listed genes represent those from our dataset that contributed to each pathway. This table serves as the source data for Supplemental Fig. S2, which visualizes the miRNA enrichment results.

**Supplemental Table. 8 Analysis of downregulated proteins.** This supplementary table presents the results of KEGG, cellular component, biological process, and molecular function enrichment analyses performed using ShinyGO (v0.85) on proteins significantly decreased in infosomes compared to conventional EVs. The table includes all enriched KEGG pathways, along with the false discovery rate (FDR), number of mapped genes from the dataset (nGenes), total number of genes annotated to each pathway (Pathway Genes), fold enrichment, and pathway name with its associated URL. The listed genes represent those from our dataset that contributed to each pathway. This dataset serves as the source data for Supplemental Fig S1.
